# mCSF-Induced Microglial Activation Prevents Myelin Loss and Promotes Its Repair in a Mouse Model of Multiple Sclerosis

**DOI:** 10.3389/fncel.2018.00178

**Published:** 2018-07-03

**Authors:** Nathalie Laflamme, Giulia Cisbani, Paul Préfontaine, Younes Srour, Jordan Bernier, Marie-Kim St-Pierre, Marie-Ève Tremblay, Serge Rivest

**Affiliations:** Neuroscience Laboratory, CHU de Québec Research Center and Department of Molecular Medicine, Faculty of Medicine, Laval University, Quebec, QC, Canada

**Keywords:** mCSF, microglial cells, multiple sclerosis, mouse models, demyelination, remyelination

## Abstract

A pathological hallmark of multiple sclerosis (MS) is myelin loss in brain white matter accompanied by compromised remyelination. Demyelinated lesions are deeply associated with oligodendrocyte apoptosis and a robust inflammatory response. Although various studies point towards a noxious role of inflammation in MS, others emphasize a positive role for the innate immune cells in disease progression. A cytokine well-known to stimulate cell survival, proliferation and differentiation of myeloid cells, macrophage colony-stimulating factor (mCSF), was administered to mice during a 5 week-long cuprizone diet. Treated mice exhibited reduced myelin loss during the demyelination phase, together with an increased number of microglia and oligodendrocyte precursor cells in lesion sites. Tamoxifen-induced conditional deletion of the mCSF receptor in microglia from cuprizone-fed mice caused aberrant myelin debris accumulation in the corpus callosum and reduced microglial phagocytic response. mCSF therefore plays a key role in stimulating myelin clearance by the brain innate immune cells, which is a prerequisite for proper remyelination and myelin repair processes.

## Introduction

Multiple sclerosis (MS) is a chronic inflammatory neurodegenerative disease of the central nervous system (CNS). A main pathological hallmark of MS is the loss of myelin sheaths protecting neuronal fibers in white and gray matter (Love, 2006; Simmons et al., 2013), leading to progressive axonal and neuronal degeneration and reduced speed of nerve conduction. While most patients exhibit an initial inflammatory relapsing-remitting pathology followed by a chronic progressive phase known as secondary progressive MS, others already present the latter phase, which is less inflammatory. Remyelination that counteracts myelin loss and prevents degeneration of the denuded axons is often compromised in chronically demyelinated lesions. Promoting remyelination while preventing demyelination is thus an important challenge in the course of finding a cure for the disease (Chang et al., 2002; Plemel et al., 2017).

Different factors act during the demyelination process. The death of oligodendrocytes, which maintain the myelin sheaths surrounding neuronal axons, is one of the first events involved in the formation of demyelinating plaques. Oligodendrocyte progenitor cells (OPCs) can migrate to the site of injury and differentiate into mature oligodendrocytes to promote the restoration of myelin sheaths. In parallel, a robust immune response, meditated by macrophages including microglia (Simmons et al., 2013), is associated with the demyelinating lesions. While a number of publications points towards a noxious role of innate immune cells in MS considering their antigen-presenting nature and ability to release pro-inflammatory cytokines, which may directly affect the myelin sheaths, others emphasize their beneficial roles in disease progression (Cash et al., 1993; Rawji and Yong, 2013; McMurran et al., 2016). Indeed, a growing body of evidence demonstrates that microglia, the resident innate immune cells of the brain, have regenerative properties, releasing growth factors and phagocytosing myelin debris during the demyelination phase (Miron et al., 2013; Rawji and Yong, 2013; Fu et al., 2014). Importantly, inhibition of macrophage activity can prevent the regenerative events that follow demyelination in animal models, notably by impeding phagocytosis (Kotter et al., 2001, 2005). Additionally, we recently established that myelin clearance is compromised in CX3CR1 knockout mice resulting in inefficient axonal remyelination (Lampron et al., 2015). An efficient myelin clearance through phagocytosis is key for proper remyelination, pointing to the critical role exerted by microglia in the demyelination/remyelination (DM/RM) process. A tight balance between boosting remyelination while limiting demyelination needs to be achieved through the modulation of oligodendrocyte survival, differentiation and proliferation, as well as microglial activity.

A number of animal models are available to mimic MS, among which the well-characterized, very reproducible and non-invasive cuprizone model allows to study cellular and molecular mechanisms involved in the DM/RM process, while excluding the autoimmune component. Indeed, continued exposition to dietary cuprizone, a copper-chelating toxin, leads to oligodendrocyte apoptosis and demyelination among vulnerable brain structures, such as the corpus callosum and cerebral cortex. Few days after replacing cuprizone by normal food, RM is observed in those structures (Matsushima and Morell, 2001; Gudi et al., 2014). Our previous study brought us to deepen the role of microglia and other parenchymal cells such as oligodendrocytes in the cascade of events that leads to DM/RM events. For this reason in this study, exogenous macrophage colony-stimulating factor (mCSF) was administered to mice that received dietary cuprizone. mCSF is a cytokine well-known to stimulate cell survival, proliferation and differentiation of myeloid cells (Hamilton, 2008; Otero et al., 2009). Moreover, it modulates microglial phenotype towards an anti-inflammatory one (Ushach and Zlotnik, 2016), reducing the expression of antigen presenting proteins (Smith et al., 2013) and promoting the release of trophic factors (Smith et al., 2013). Finally, it also regulates the phagocytic activity of microglia. Indeed, we previously reported an improved clearance of amyloid beta in a mouse model of Alzheimer’s disease upon treatment with mCSF (Boissonneault et al., 2009). Herein, we investigated the effect of exogenous mCSF on the activity of microglia and oligodendrocytes over the course of cuprizone intoxication. Since mCSF is expressed constitutively, we utilized a conditional model in which its mCSF receptor (CSF1R) is deleted in microglia selectively, to better understand the role of the endogenous cytokine.

## Materials and Methods

### Animal Care

One-hundred and forty-five 7-week-old male C57BL/6j mice were used in this study. All animals were acclimated to standard laboratory conditions with *ad libitum* access to mouse chow and water. Mice were housed up to four per cage (ventilated cages from Lab product) in temperature and light-controlled room (12 h-light cycle from 7 am to 7 pm) and were fed and allowed to drink water *ad libitum*. The health status of all mice was monitored throughout the experimental protocol, recording weight loss and any other signs of health-related issues. All protocols were performed in accordance to the Canadian Council on Animal Care guidelines, as administered by the Laval University Animal Welfare Committee. All experiments were approved by the committee.

### Conditional Csf1Rko Mice

B6.Cg-Csf1r *tm1jwp*/J mice (JaxMice; stock number 02212) were crossed with the B6.129-Cx3cr1tm2.1(CreER)Jung/Orl mice (EMMA mouse respiratory; EM:06350). The resulting mouse has a tamoxifen-inducible CRE activity specifically in microglial cell, leading to a non-functional CRFR1 protein. Five days after the last tamoxifen administration (5 mg/day for 4 days by gavage), mice were fed with normal or cuprizone-supplemented chow for 5 weeks.

### Chimeric Mice

Experimental animals received a total of 80 mg/kg of Busulfan administered i.p. every 12 h for 4 days, followed by 2 days of single i.p. injection of 100 mg/kg cyclophosphamide. After a 24-h rest, 3 × 10^7^ bone marrow cells isolated from the tibia and femur of donor mice were injected into the tail vein of target animals. C57BL/6-Tg(CAG-EGFP)1O sb/J (JaxMice stock number 003291) mice were used as donors. For details on this procedure, please refer to previous studies (Lampron et al., 2012).

### Cuprizone Diet and mCSF Treatment

**Figure 1:**
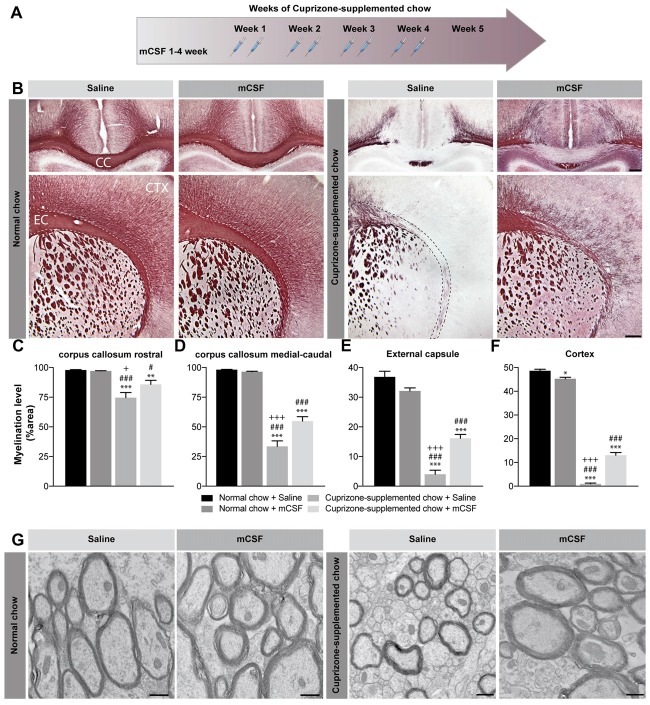
Effect of mCSF administration on cuprizone-induced demyelination. **(A)** Timeline of mCSF administration during the 5 weeks cuprizone intoxication. **(B)** Representative photomicrographs showing myelin staining (Black Gold II) in the medial corpus callosum (upper images) and in the external capsule and cortex (lower images) of mice fed with normal or cuprizone-supplemented chow for 5 weeks and injected two times/week with mCSF (40 μg/kg) or saline (0.9%) during the first 4 weeks of diet. **(C–F)** Quantification of myelination level, measured as percentage of area occupied by the staining, measured in the corpus callosum **(C,D)**, external capsule **(E)** and cortex **(F)** of mCSF- or saline-treated animal fed with cuprizone or normal diet. **(G)** Representative electron microscopy image showing myelin integrity in the corpus callosum of cuprizone-fed mice injected with either saline or mCSF compared to animals fed with normal food. Values are expressed as means ± standard error of the mean (SEM). Statistical analyses were performed using a two-way ANOVA followed by a Tukey’s multiple comparisons test. *p < 0.05, **p < 0.005, ***p < 0.001 significance different compared to the group that received the normal chow + Saline; ^#^*p* < 0.05, ^###^*p* < 0.001 significance different compared to the group that received the normal chow + mCSF; ^+^*p* < 0.05, ^+++^*p* < 0.001 significant difference compared to the group that received cuprizone-supplemented chow + mCSF. *n* = 6–8 mice/group. Scale bar: **B**: 150 μm; **G**: 500 nm. Abbreviations: CC, corpus callosum; CTX, cortex; EC, external capsule; mCSF, macrophage colony stimulating factor.

**Figure 2:**
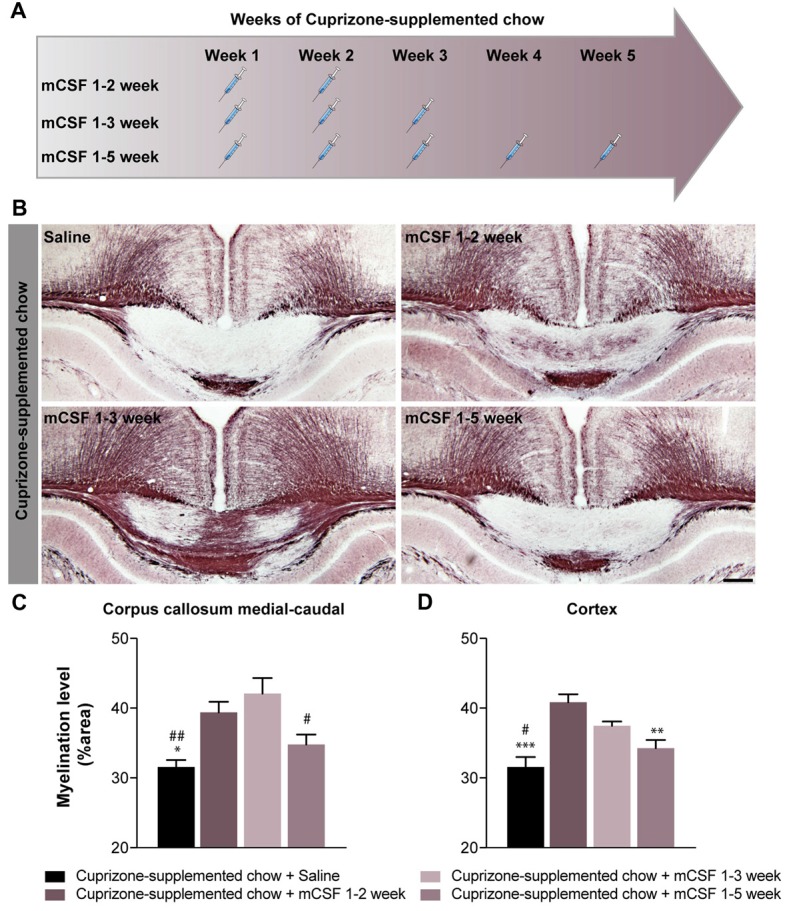
The beneficial effects of mCSF is influenced by the schedule of administration in cuprizone-fed mice. **(A)** Timeline of mCSF administration during the 5 weeks cuprizone intoxication. **(B)** Representative photomicrographs depicting myelin staining (Black Gold II) in the corpus callosum of cuprizone-fed mice and injected with either saline 0.9% or mCSF (40 μg/kg) following different administration schedule **(A)**. **(C,D)** Graphs represent the myelination level, expressed as the percentage of area occupied by the staining, in the corpus callosum **(C)** and cortex **(D)**. Values are expressed as means ± SEM. Statistical analyses were performed using a one-way ANOVA followed by a Tukey’s multiple comparisons test. *p < 0.05, **p < 0.005, ***p < 0.001 significance different compared to the group that received the cuprizone-supplemented chow + mCSF 1–2 week ; ^#^*p* < 0.05, ^##^*p* < 0.001 significance different compared to the group that received the cuprizone supplemented chow + mCSF 1–3 week. *n* = 5–8 mice. Scale bar: **B**: 150 μm. Abbreviations: mCSF, macrophage colony stimulating factor.

0.2% wt/wt cuprizone (bis-cyclohexylidene hydrazide; Sigma-Aldrich) was mixed with regular ground irradiated chow and fed to experimental animals for 5 weeks. The chow was changed every 2 days and food intake was monitored throughout the protocols. Control animals were fed with regular irradiated ground chow and manipulated as often as cuprizone-fed mice. After terminating the 5 weeks of cuprizone diet, a group of mice (*n* = 16) was kept for additional 5 days on regular ground chow. Concomitantly to the first 4 weeks of cuprizone-rich diet, mice were injected twice a week with either mCSF diluted in saline (40 μg/kg) or vehicle (saline 0.9%; see Timeline Figure 1A). A group of mice (*n* = 40) were injected i.p. once a week with either mCSF diluted in saline (40 μg/kg) or vehicle during either the first two (mCSF 1–2 week) or 3 weeks (mCSF 1–3 week) of diet or during the entire duration of the diet (mCSF 1–5 week; see Timeline Figure 2A). After 5 weeks of cuprizone-supplemented diet, another group of mice (*n* = 29) was fed normal chow and was treated for an additional week with mCSF or saline (see Timeline Supplementary Figure S3A).

### Sacrifices

After 5 weeks of diet, all mice were deeply anesthetized with ketamine/xylazine and sacrificed via intracardiac perfusion with 0.9% saline followed by 4% PFA pH 7.4. The brains were then retrieved, post-fixed 10–24 h in 4% PFA pH 7.4 and transferred in 4% PFA pH 7.4 + 20% sucrose for a minimum of 15 h. Brains were sliced in coronal sections of 25-μm thickness with a freezing microtome (Leica Microsystems), serially collected in anti-freeze solution and kept at −20°C until usage. Three mice from all groups were perfused with 3.5% acrolein (in 100 mM phosphate buffer [PB], pH 7.4) followed by 4% paraformaldehyde pH 7.4 for electron microscopy. Longitudinal sections of the brain (50 μm thick) were cut with a vibratome in ice-cooled PBS (0.9% NaCl in 50 mM phosphate buffer, pH 7.4).

## Post-Mortem Analyses

### Black Gold Staining and Quantification of Myelination

Brain sections collected as described above were washed three times for 10 min in cold KPBS and mounted onto Micro Slides Superfrost plus (VWR International). The slides were pre-warmed 30 min at 65°C on a slide warmer, washed once with warm KPBS, followed by an incubation in 0.3% Black Gold II (EMD Millipore) diluted into 0.9% NaCl for 30 min. After this time, slides were washed in warm KPBS and then in warm sodium thiosulfate for 3 min and then transferred into KPBS. All the steps were performed at 65°C. Finally, the slides were dehydrated in alcohol, cleared in xylene and coverslipped with DPX.

### Immunohistochemical Staining

Brain sections were washed (4 × 5 min) in KPBS and then blocked in KPBS containing 1% BSA, 4% NGS and 0.4% Triton X-100. The tissues were then incubated overnight at 4°C with the primary antibody anti-Iba-1 (rabbit, 1:1000; DAKO) and anti-Olig2 (rabbit, 1:1000; Millipore). After washing the section in KPBS (4 × 5 min), tissues were incubated in the appropriate secondary antibody (biotinylated goat anti-rabbit IgG; 1:1500, Vector Laboratories) for 2 h at room temperature. Following further washes in KPBS and 1 h-long incubation in avidin–biotin peroxidase complex (ABC; Vector Laboratories) the sections were then incubated in 3,3′-diaminobenzidine tetrahydrochloride (DAB; Sigma) to reveal the staining. The sections were mounted onto Micro Slides Superfrost plus glass slides, dehydrated and then coverslipped with DPX mounting media.

### Immunofluorescent Staining

Brain sections were washed (4 × 5 min) in KPBS then blocked in KPBS containing 1% BSA, 4% NGS and 0.4% Triton X-100. An antigen retrieval step was performed to stain for Ki67. More specifically, sections were boiled 10 min in sodium citrate 10 mM pH 6 just before the blocking step. The tissues were then incubated overnight at 4°C with the primary antibody anti-Ki67 (mouse, 1:200; BD Bioscience), anti-Olig2 (rabbit, 1:1000; Millipore) or anti-CD68 (rat, 1:750; Serotec). After washing the section in KPBS (4 × 5 min), the tissue was incubated in the appropriate secondary antibody (IgG anti-rabbit CY3; Jackson Immunoresearch and IgG anti-mouse alexa 488; Thermofisher) for 2 h at room temperature. After the staining for CD68, sections were incubated 30 min in 0.5 mM 1,1’-Dioctadecyl-3,3,3’,3’-tetramethylindocarbocyanine perchlorate or DiI (Sigma-Aldrich) in the blocking solution (Lampron et al., 2015). Following further washes in KPBS and incubation with DAPI to identify the nuclei, the sections were mounted onto Micro Slides Superfrost^®^ Plus glass slides and coverslipped with Fluoromount-G (Electron Microscopy Sciences).

## *In Situ* Hybridization

### Free-Floating

One every 12th sections of the whole rostrocaudal extent of each brain was mounted on Micro Slides Superfrost Plus. Prehybridization step including digestion with proteinase K were performed to prepare the section for hybridization as previously reported (Laflamme et al., 1999).

### Hybridization

Histochemical localization of each mRNA transcript was performed using ^35^S-labeled cRNA probes encoding toll-like receptor 2 (*TLR2*), triggering receptor expressed on myeloid cells 2 (*TREM2*), platelet-derived growth factor receptor alpha (*PDGFRα*) and Insulin-like growth factor 1 (*IGF-1*) were performed using ^35^S-labeled cRNA probes (Laflamme and Rivest, 2001; Lampron et al., 2015). Plasmids were linearized and antisense cRNA probes were synthesized with an appropriate RNA polymerase. All plasmids were analyzed for sequence confirmation and orientation. Tissue slides were hybridized with the probe overnight at 65°C on a slide warmer. After post-hybridization treatment, gene expression was revealed by placing sections covered by a Biomax X-ray film (Kodak) in an autoradiography cassette (Fisher Scientific) for 3 days. Films were then scanned using an Epson Perfection v850 Pro scanner supported by the SilverFast software (version 8.8.Or6). Area and intensity of positive hybridization signals were densitometrically measured on all brain sections using ImageJ software (Version 2.0.0-rc-43/1.51n). Each value was corrected for background signal by subtracting the OD value measured at a brain area devoid of positive signal (for a detailed protocol see Laflamme et al., 1999).

## Electron Microscopy and Analyses

The tissue for electron microscopy was prepared according to previously published protocol (Lampron et al., 2015) and examined with an FEI Tecnai G2 Spirit BioTwin electron microscope. Imaging and analysis were conducted blind to the experimental conditions. Oligodendrocytes were recognized by their dark cytoplasm, generally rectangular shape of nucleus, enlarged space between nuclear membranes, distinctive short stretches of endoplasmic reticulum and direct association with myelin sheaths (Peters et al., 1991). In addition, apoptotic oligodendrocytes had a pyknotic nucleus showing chromatin fragmentation and/or condensation, and frequently contained lipofuscin granules as well as autophagocytic vacuoles and small spherical bodies (Tinari et al., 2008).

### Image Acquisition and Analyses

Image acquisition of Fluorescent staining images was performed using a Zeiss LSM800 confocal microscope supported by the Zen software (2.3 system) using the 10× and 40× lenses. Confocal images were then processed using Fiji (ImageJ Version 2.0.0-rc-43/1.51n). For analyses and brightfield image acquisition of the Black Gold II staining, Iba-1 and Olig2, 8-bit grayscale TIFF images of the regions of interest were taken in a single sitting for whole protocols with a Qimaging camera (Qcapture program, version 2.9.10), attached to Nikon microscope (C-80) with the same gain/exposure settings for every image. To evaluate the level of myelination and Iba-1^+^ immune response in the regions of interest (cortex, corpus callosum and external capsule), the images were imported into ImageJ (1.37) and the percentage of area occupied by the staining was measured using the threshold parameter. On the other end, Olig2^+^ cells were manually counted with ImageJ. Fluorescent staining of images was performed using a Zeiss LSM800 confocal microscope supported by the Zen software (2.3 system). Confocal images were then processed using Fiji (ImageJ Version 2.0.0-rc-43/1.51n).

## Statistical Analyses and Figure Preparation

Data are presented as mean ± standard error of the mean (SEM). Statistical analyses were carried with the Prism software (version 6.0, GraphPad Software Inc.). Values were considered statistically significant if *p* < 0.05. All panels were assembled using Adobe Photoshop CS5 (version 12.0.4) and Adobe Illustrator CS5 (version 15.0.2).

## Results

### Cuprizone Diet Reduces Body Weight in Wild Type Mice

Wild type mice were fed with normal chow or cuprizone-supplemented chow during 5 weeks, as the peak of demyelination occurs between 4 weeks and 5 weeks of diet. After this period, the phase of remyelination begins, overlapping with the demyelination one (Gudi et al., 2014; Vega-Riquer et al., 2017). During the first 4 weeks of diet, mCSF (40 μg/kg) or saline was administered intraperitoneally twice a week. Mice were followed-up throughout the experimental course to evaluate food intake as well as body weight. Although we did not observe differences in food intake among groups (Supplementary Figure S1A), both groups fed with cuprizone-supplemented chow presented weight loss (about 8% of initial body weight, Supplementary Figure S1B) as expected (Stidworthy et al., 2003; Steelman et al., 2012). These results indicate that weight loss resulted from the dietary cuprizone, instead of malnutrition.

### mCSF Treatment Reduces Demyelination in Cuprizone-Intoxicated Mice

Continued cuprizone exposure during a 5-week period induces a consistent demyelination in brain white matter, affecting vulnerable regions such as corpus callosum, external capsule and cortex (Stidworthy et al., 2003; Wu et al., 2008; Xie et al., 2010; Steelman et al., 2012). Indeed, Black Gold II immunostaining depicts a specific profile of demyelination (Figure 1B), with a rostro-caudal progression of the pathological features in the corpus callosum that is particularly susceptible to the drug. As expected, more severe myelin depletion is detected in the medial-caudal area of the corpus callosum, while the external capsule is most affected rostrally (Figures 1C–E and Supplementary Figure S2A; Stidworthy et al., 2003; Wu et al., 2008; Xie et al., 2010). Cortical myelin fibers are also susceptible to the cuprizone dietary regimen (Figure 1F). Importantly, mCSF administration restrained this loss and myelin fibers occupied a more extended area of the rostral (Figures 1B,C and Supplementary Figure S2A) and medial-caudal corpus callosum (Figures 1B,D and Supplementary Figure S2A). Similarly, both the external capsule (Figures 1B,E) and cortex (Figures 1B,F) displayed larger myelin coverage. Noteworthy, dense myelin staining was observed in normal chow-fed mice (Figures 1B–F). For a more detailed evaluation of myelin ultrastructure in the corpus callosum, electron microscopy observations were conducted revealing that the axons of the cuprizone-fed mice are largely demyelinated, showing dystrophy with an accumulation of autophagocytic vacuoles in the myelinic sheaths, and a smaller diameter compared to those of normal-chow fed mice (Figure 1G). mCSF administration during a 5 week-period of cuprizone diet improved the overall organization of the myelin sheaths, resulting in axons resembling those of the control group (Figure 1G). Since the corpus callosum is the largest white matter region of the brain (Fitsiori et al., 2011), easily defined and highly susceptible to dietary cuprizone, the majority of results herein presented refer to the medial-caudal callosal area. Overall, these findings suggest beneficial effects of mCSF treatment during the demyelination phases in reducing myelin loss in the brain of cuprizone-intoxicated mice.

### mCSF Administration Limited to the Early Phases of Demyelination Is Sufficient to Reduce Myelin Depletion in Cuprizone-Intoxicated Mice

Persistent stimulation of the immune response has been shown to be detrimental in MS (Yong, 2010). Thus, we explored whether a reduced number of mCSF injections, administered during the earlier phase of demyelination in a 5-week period of cuprizone regimen would still be beneficial. Indeed, when mCSF was limited to the first 2 (mCSF week 1–2) or 3 weeks (mCSF week 1–3) of diet (see timeline Figure 2A), myelin coverage was restored to a larger extent in the medial-caudal corpus callosum and cerebral cortex (Figures 2B–D) compared to both the control and mCSF administration during the entire 5 weeks of cuprizone-supplemented diet (mCSF week 1–5). These results indicate that acute/punctual treatment with mCSF at the onset of demyelination is sufficient to generate long-term effects in the brain of cuprizone-intoxicated mice, while administration of the drug at later stages, when the cuprizone diet was terminated, has no effect (Supplementary Figure S3).

### mCSF Boosts Microglial Activity in Cuprizone Mouse Model

**Figure 3:**
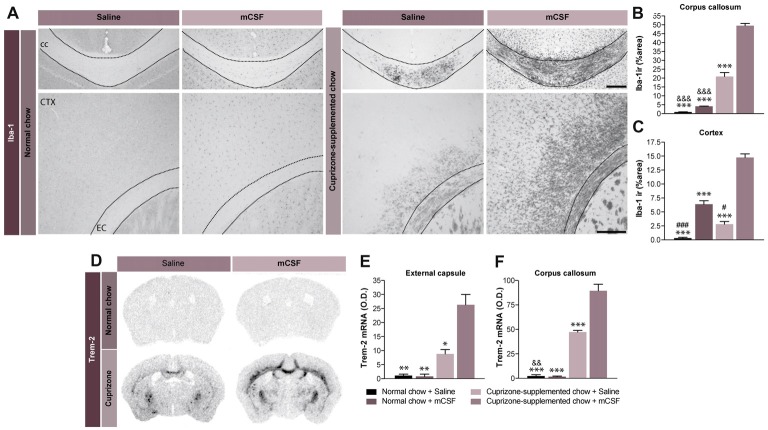
Potential beneficial effect of microgliosis induced by mCSF. **(A)** Representative photomicrographs of Iba-1+ immunostaining in the corpus callosum and the cortex of mice fed with normal or cuprizone-supplemented food and injected twice a week with mCSF (40 μg/kg) or saline during the first 4 weeks of the diet. **(B,C)** Graphs represent the % of the area covered by Iba-1 staining in the corpus callosum **(B)** and cortex of the treated mice **(C)**. **(D–F)** Representative images of *TREM-2* mRNA expression (*in situ* hybridization) and the respective quantification of the expression levels of *TREM-2* mRNA signal measured in the external capsule **(E)** and corpus callosum **(F)** of treated mice. Values are expressed as means ± SEM. Statistical analyses were performed using a two-way ANOVA **(B,C)** followed by a Tukey’s multiple comparisons test. *p < 0.05; **p < 0.005; ***p < 0.001 significance different from the group that received the cuprizone-supplemented chow + mCSF; ^&&^*p* < 0.005, ^&&&^*p* < 0.001 significance different from the group that received the cuprizone-supplemented chow + Saline; ^#^*p* < 0.05, ^###^*p* < 0.001 significance different from the group that received the normal chow + mCSF. *n* = 3–8 mice per group. Scale bar: **A**: 150 μm. Abbreviations: cc, corpus callosum; CTX, cortex; Iba-1 ir, ionized calcium binding adaptor molecule 1 immunoreactive signal; mCSF, macrophage colony stimulating factor; TREM2, Triggering receptor expressed on myeloid cells 2.

Concomitant to the demyelination process, activated microglia can be discerned in the brain of cuprizone-exposed mice. Importantly, microglia are strategically located in regions of strong demyelination (Matsushima and Morell, 2001) where they contribute to the clearance of myelin debris, a role essential for efficient remyelination (Lampron et al., 2015). Consistently with our previous report (Lampron et al., 2015), microgliosis defined by an increased immunoreactivity for Iba-1 was found in cerebral areas associated to myelin loss, including the cerebral cortex, corpus callosum (Figures 3A–C), and external capsule (data not shown), 5 weeks post-dietary cuprizone. Treatment with mCSF further boosted such microgliosis (Figures 3A–C). Additionally, mCSF increases the expression of *TREM2* mRNA in the external capsule and corpus callosum, suggesting that it increases phagocytic activity in cuprizone-intoxicated mice (Figures 3C–F). This further points to the contribution of the immune response in clearing myelin debris.

### Modulation of Immune Response Leads to an Increased Expression of IGF-1 That May Contribute to the Beneficial Effects of mCSF

**Figure 4:**
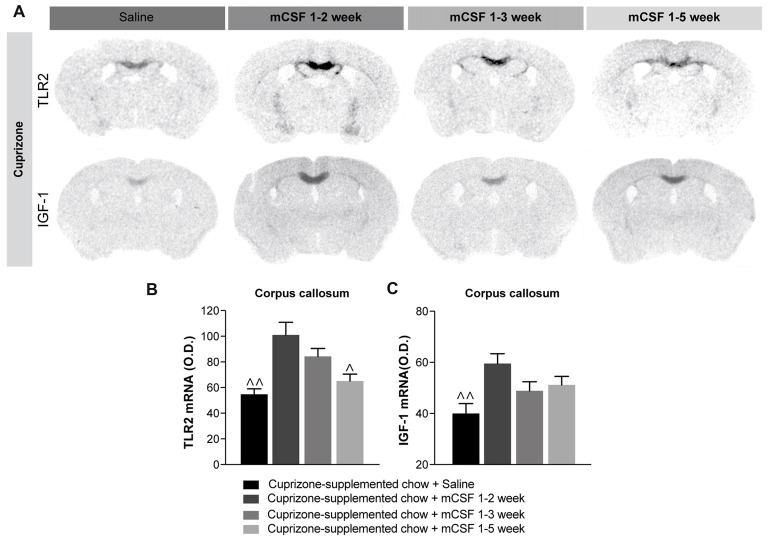
Administration of mCSF in the early phase of disease progression is beneficial for remyelination. **(A)** Representative images of TLR2 and *IGF-1* mRNA expression (*in situ* hybridization) and **(B,C)** corresponding graphs showing the semi-quantitative levels of the two transcripts in the corpus callosum of mice on a 5 weeks cuprizone-supplemented diet or a normal chow diet and injected with saline or mCSF (40 μg/kg) according to the injection schedule of Figure 2A. Values are expressed as means ± SEM. Statistical analyses were performed using one-way ANOVA followed by a Tukey’s multiple comparisons test. ^∧^*p* < 0.05, ^∧∧^*p* < 0.01 significance different from the group that received the cuprizone-supplemented chow + mCSF 1–2 week. *n* = 3–8 mice per group. Abbreviations: IGF-1, insulin-like growth factor1; O.D., Optical Density; TLR2, toll-like receptor 2.

A connection between inflammation and remyelination has been previously established (Rawji and Yong, 2013; Wlodarczyk et al., 2015; McMurran et al., 2016). Indeed, stimulation of the neuroinflammatory response might promote the recruitment of OPCs, accelerating the remyelination process (Glezer et al., 2006). mCSF treatment modulates the parenchymal immune response by increasing expression of the inflammatory toll-like receptor 2 (TLR2) on microglial cells (Laflamme et al., 2003; Kigerl et al., 2007). More specifically, increased *TLR2* gene expression levels were measured (Figures 4A,B) when mCSF was limited to the first 2 (mCSF week 1–2) or 3 weeks (mCSF week 1–3) of diet (see timeline Figure 2A). Interestingly, *TLR2* gene expression was higher and persisted to the end of diet when the cytokine was administered during the first 2 weeks of the cuprizone-supplemented diet compared to mice receiving either saline or mCSF along the 5 weeks of diet. Concomitantly to the increased microglial activation, the expression level of the trophic factor *IGF-1*, suggested to govern the remyelination process in the cuprizone model (Mason et al., 2000a), remained upregulated following only two injections of mCSF (Figures 4A–C). This trophic factor thus appears to play a key role in promoting the recovery and survival of oligodendrocytes.

### mCSF Promotes the Recruitment of Oligodendrocyte Progenitor Cells in the Corpus Callosum of Cuprizone-Intoxicated Mice

**Figure 5:**
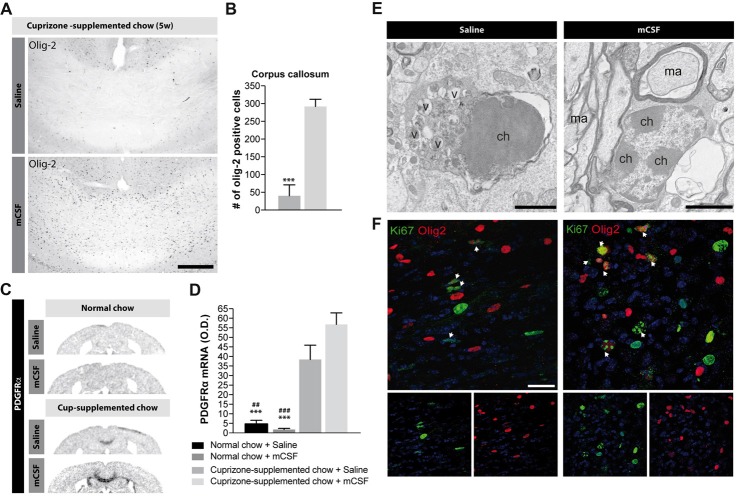
Effects of mCSF on the number of oligodendrocytes and oligodendrocyte progenitors in demyelinated regions of cuprizone-treated mice. **(A,B)** Representative photomicrographs of Olig2-immunoreactive staining **(A)** and their respective quantification in the corpus callosum **(B)** of mice fed with cuprizone-supplemented chow for 5 weeks and injected twice a week with either saline or mCSF (40 μg/kg) during the first 4 weeks of diet. **(C,D)** Representative micrographs of PDGFRα mRNA expression and semi-quantitative expression level in the corpus callosum **(D)** in mice fed with normal or cuprizone-supplemented chow and injected with either saline or mCSF. **(E)** Representative electron microscopy images showing apoptotic vs. healthy oligodendrocytes in the corpus callosum of mice on a 5 weeks cuprizone diet injected with saline or twice a week with mCSF (40 μg/kg), respectively. **(F)** Confocal images showing co-localization of Olig2 positive cells (red) with Ki67 immunoreactive cells (green) in the corpus callosum of mice fed with cuprizone-supplemented diet and treated with saline or mCSF. White arrows point-out some examples of co-localization. Values are expressed as means ± SEM. Statistical analyses were performed using a Mann-Whitney *t*-test **(B)**, and two-way ANOVA followed by a Tukey’s multiple comparisons test **(D)**. ***p < 0.001 significantly different from the group that received the cuprizone-supplemented chow + mCSF; ^##^*p* < 0.005, ^###^*p* < 0.001 significantly different from the group that received the cuprizone-supplemented chow + saline. *n* = 3–8 mice. Scale bar: **A**: 150 μm; **E**: 2 μm; **F**: 25 μm. Abbreviations: Cup, cuprizone; O.D, optical density; v, autophagic vacuole; ch, chromatin; ma, myelinated axon; mCSF, macrophage colony stimulating factor; Olig2, oligodendrocyte transcription factor 2; PDGFRα, platelet-derived growth factor receptor alpha.

While cuprizone diet results in demyelination, OPCs proliferate and differentiate to re-establish the myelin sheaths on denuded axons (Sachs et al., 2014). mCSF treatment elicits a considerable increase in the number of Olig2^+^ oligodendrocytes in the corpus callosum, compared with the control group (Figures 5A,B). This result was corroborated by the slight increased expression level (*p* = 0.07) of a marker of oligodendrocyte progenitors *PDGFR*α following mCSF treatment (Figures 5C,D). Importantly, ultrastructural analyses in corpus callosum of mice that underwent cuprizone intoxication revealed that oligodendrocytes are often in apoptosis, presenting nuclear heterochromatin condensation, dystrophic morphologies, and an accumulation of autophagocytic vacuoles, while oligodendrocytes of mice treated with mCSF rarely show such degenerative features (Figure 5E). Finally, co-localization of Olig2^+^ cells with the proliferation marker Ki67 indicated that more oligodendrocytes are in proliferating status upon mCSF administration (Figure 5F). Spontaneous remyelination occurs when the mice are returned to the normal diet following the cuprizone-rich diet (Matsushima and Morell, 2001) and this process is also accompanied by an increased proliferation of Olig2^+^ cells (Supplementary Figure S4A). mCSF treatment further contributed to increasing this proliferation (Supplementary Figures S4A,B). These results suggest that mCSF promotes earlier proliferation and better survival of oligodendrocytes in a demyelination paradigm.

### mCSF Does Not Affect Infiltration of Peripheral Immune Cells, While Triggering Cell Proliferation

Although circulating myeloid cells are greatly recruited at the lesion site as reported previously (McMahon et al., 2002; Lampron et al., 2015), mCSF treatment does not affect peripheral GFP^+^ cell infiltration in cuprizone-intoxicated chimeric mice generated with a chemotherapeutic conditioning (GFP → WT). In fact, the number of GFP^+^ cells is similar in both treated and non-treated mice (Supplementary Figure S5A–C). However, mCSF seems to promote cell proliferation as an increased level of Ki67+ cells were observed in the corpus callosum of mCSF injected mice compared to their control counterparts (Figure 5F). Importantly microglia proliferation outnumbers the infiltrating cells suggesting that the microglia cells might play a central role in the process of remyelination.

### Knocking-Out mCSF Receptor Selectively on Microglia Affect Myelin Clearance

**Figure 6:**
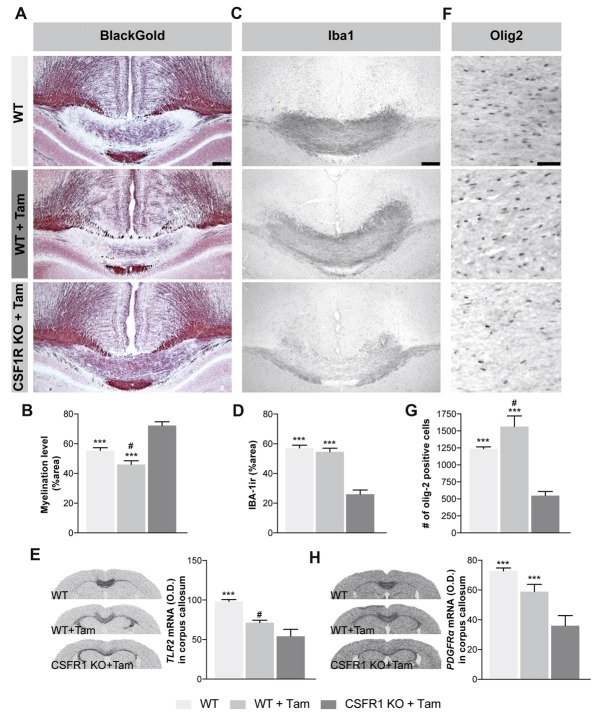
Selective absence of mCSF receptor in microglial cells leads to an inefficient OPCs production and aberrant myelin patterns following cuprizone-supplemented diet. **(A)** Representative photomicrographs show myelin staining (Black Gold II) in the medial corpus callosum of CSF1R-CX3CR1CreErt mice that received tamoxifen (5 mg/day for 4 days by gavage) and that underwent 5 week-long cuprizone-supplemented diet. **(B)** Quantification of demyelination, presented as percentage of area occupied by the staining, measured in the corpus callosum. **(C)** Representative photomicrographs of Iba-1-immunoreactive staining **(D)** and their respective quantification in the corpus callosum. **(E)** Representative images of *TLR2* mRNA expression (*in situ* hybridization) and the respective quantification of their expression levels measured in the corpus callosum. **(F)** Representative photomicrographs of Olig2-immunoreactive staining and **(G)** their respective quantification in the corpus callosum. **(H)** Images of *PDGRα* mRNA expression level (*in situ* hybridization) and its respective quantification. Values are expressed as means ± SEM. Statistical analyses were performed using an one-way ANOVA followed by a Tukey’s multiple comparisons test. ***p < 0.001 significantly different from the group CSFR1 KO + Tam; ^#^*p* < 0.05 significantly different from the group WT. *n* = 3–6 mice. Scale bar: **A,C,F**: 150 μm. Abbreviations: Iba-1 ir, ionized calcium binding adaptor molecule 1 immunoreactive signal; Olig-2, oligodendrocyte transcription factor 2; TLR2, toll-like receptor 2.

The results presented so far show that peripheral exogenous administration of mCSF limits the demyelination process induced by dietary cuprizone, polarizing microglial cells towards the lesion site, while promoting their phagocytosis and release of the growth factor IGF-1. We further dissected the role of endogenous mCSF microglial signaling in adult mice undergoing cuprizone intoxication, by utilizing a conditional knockout mouse model in which the mCSF receptor CSF1R, is selectively deleted in microglia (Yona et al., 2013; Peng et al., 2016; Fonseca et al., 2017). Following a cuprizone-enriched diet, CSF1R-loxP-CX3CR1-cre/ERT2 mice exposed to tamoxifen presented a higher level of myelin staining in the corpus callosum and other regions normally affected by cuprizone (Figures 6A,B), accompanied by a reduced microgliosis (Figures 6C,D). Importantly, the inflammatory activity of microglia, determined by the level of TLR2 mRNA, was lower in the knockout mice (Figure 6E). Finally, we observed a reduced number of Olig-2-positive OPCs in the corpus callosum of cuprizone intoxicated knockout mice (Figures 6F,G), along with a lower expression level of *PDGFRα* (Figure 6H). These results suggest that CSF1R signaling in microglia plays a critical role in the demyelination phase and that myelin elimination can interfere with the mechanisms involved in the remyelination processes as Olig2-positive cells proliferation is reduced in these mice.

**Figure 7:**
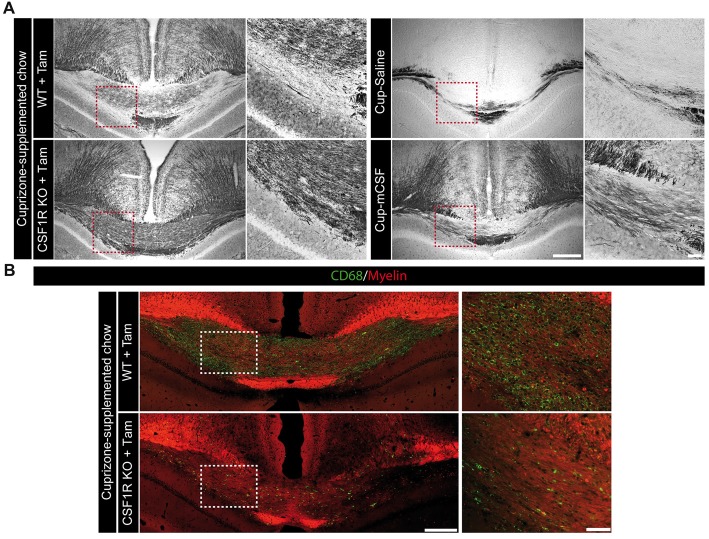
The absence of microglial mCSF receptor prevents the proper clearance of myelin debris following cuprizone-supplemented diet. **(A)** Representative photomicrographs showing the myelin status (Black Gold II) in the medial corpus callosum of CSF1R-loxP-CX3CR1-cre/ERT2 mice that received tamoxifen (5 mg/day for 4 days by gavage) and that underwent 5 week-long cuprizone-supplemented diet as well as cuprizone-intoxicated wild type mice treated with saline or mCSF. High power magnifications showing a representative area of the corpus callosum, identified by the red dotted-line squares. **(B)** Representative photomicrographs of CD68 (green) and myelin (0.5 mM 1,1-Dioctadecyl-3,3,3,3-tetramethylindocarbocyanine perchlorate (DiI); red) staining in the corpus callosum of mCSF receptor knock-out brains along with higher power magnifications of the region highlighted by white dotted-line squares. Scale bar: **A,B**: 200 μm; insets: 50 μm. Abbreviations: Cup, cuprizone; mCSF, macrophage colony stimulating factor; Tam, tamoxifen.

Although a greater myelin coverage was observed in the corpus callosum of the CSF1R knock-out mice compared to their controls (Figures 6A,B), a careful examination of the myelin fibers at higher power magnification (Figure 7A) revealed an aberrant myelin pattern in the knock-out mice after a cuprizone-supplemented diet. On the contrary, mCSF-treated mice presented a better clearance of the myelin debris as well as an improved recovery of the fibers (Figure 7A). The burden of aberrant myelin debris found in the corpus callosum of CSF1R knockout mice likely results from the limited phagocytic activity of brain immune cells as observed with the immunostaining for CD68, a lysosomal marker indicative of the phagocytic activity of microglial cells (Taylor et al., 2005; Safaiyan et al., 2016), Indeed, the corpus callosum is nearly devoid of CD68-positive cells following tamoxifen exposure in CSF1R-loxP-CX3CR1-cre/ERT2 mice (Figure 7B). Therefore, the selective depletion of CSF1R in microglia does not only affect their ability to proliferate but also to act as proper phagocytic cells. Importantly, microglia are actively implicated in the clearance of myelin debris (Safaiyan et al., 2016), which promotes a regenerative environment that favors proper remyelination in the lesion area (Neumann et al., 2009). Thus, our results suggest that microglial CSFR1 could play a critical role in remyelination process as its absence prevents the proper clearance of myelin debris.

## Discussion

Continued exposure to dietary cuprizone induces primary demyelination in adult mice, mimicking the myelin loss observed in MS patients, especially those suffering from primary and secondary progressive symptoms. Thus, we exploited this model to test the effect of exogenously administered mCSF in cuprizone-intoxicated mice during the demyelination phase. Cuprizone is a copper-chelator agent that given at low concentration impairs oligodendrocyte viability without affecting other cell types (Hiremath et al., 1998). Mature oligodendrocytes are depleted by apoptosis during cuprizone intoxication in adult mice while OPCs proliferate and are recruited to the site of lesion in a time course overlapping with the active demyelination to regenerate the myelin around denuded axons (Döring et al., 2015). Additionally, massive microgliosis is usually observed at the site of demyelination in the third week of diet, correlating with the progression of demyelination (Hiremath et al., 1998). This pool of phagocytic cells at the site of lesion might be crucial for clearing the myelin debris during demyelination. We observed a more extended myelin coverage in the brain together with an increased immune response in the corpus callosum following mCSF administration. The cytokine also modulated the proliferation of oligodendrocytes measured by the increased number of Olig2^+^ cells and *PDGFR*α expression levels in the corpus callosum of treated mice. To further understand the role of mCSF in the demyelination/remyelination process, we used conditional knockout mouse model in which the mCSF receptor, CSFR1, is selectively deleted in microglia. At the end of the cuprizone-supplemented diet, these animals presented a heavy myelin debris burden in the corpus callosum along with a reduced number of microglia and OPCs when compared to their controls. These data allow us to propose that stimulation of mCSF/CSFR1 pathway in microglia is crucial for the clearance of degenerated myelin elements, which is required to set the proper conditions for repair.

Although often seen as a noxious event in MS, the robust immune response associated with the demyelinating plaques could be modulated towards a protective phenotype using the cytokine mCSF. This would counterbalance the demyelination process while promoting remyelination. Importantly, a number of publications point towards the connection between inflammation and remyelination. OPCs are often associated with a robust presence of macrophages/microglia as reported in human post mortem studies of MS patients (Wolswijk, 2002). However, the general opinion consider inflammation detrimental, advocating the use of anti-inflammatory drugs in MS. Nonetheless, when minocycline (Tanaka et al., 2013) or dexamethasone (Glezer et al., 2006) were given to inactivate microglia following dietary cuprizone or in other toxic models of demyelination, the treatment decreased the number of oligodendrocytes, prevented myelin recovery (Tanaka et al., 2013) and enlarged the lesion site (Glezer et al., 2006). On the contrary, administration of the endotoxin lipopolysaccharide (LPS) in demyelinated mice improved the outcome suggesting that an inflammatory response might be beneficial for remyelination (Glezer et al., 2006). However, LPS is unsafe in human so the use of approved drugs is recommended while modulating the microglial/macrophages response and their phagocytic activity. In this regard, mCSF may be a strong candidate to facilitate remyelination by clearing the myelin debris.

mCSF is a cytokine known to promote the recruitment of macrophages inducing the release of CCL2, a chemokine mobilizing monocytes to sites of inflammation (Ushach and Zlotnik, 2016). Additionally, it induces microglia/macrophage proliferation and the polarization of these cells towards a M2-like phenotype, impairing their ability to release pro-inflammatory factors and toxic mediators, while favoring the release of mediators promoting tissue repair (Hamilton, 2008; Martinez and Gordon, 2014). Mice that have null mutation for mCSF show a reduced number of tissue macrophages, whereas local expression of mCSF can rescue macrophage development indicating its crucial involvement in their homeostasis (Ryan et al., 2001). Another important player is the mCSF receptor CSFR1, which is highly expressed on monocytes and macrophages (Stanley and Chitu, 2014). Administering mCSF to cuprizone-fed mice, we identified potential mechanisms that acting in concert might be protective in the demyelination phase and promote remyelination. More specifically, mCSF cascade might stimulate the survival/proliferation of oligodendrocytes, enhancing the immune response as well as modulating the release of growth factors, such as IGF-1 and the phagocytic activity of immune cells to remove myelin debris.

In order to counterbalance the demyelination process that occurs during cuprizone intoxication, mCSF may promote the survival of the existing oligodendrocytes but most importantly, the proliferation of OPCs, marked with Olig2 and PDFGRα and their migration to the lesion area. Endogenous proliferative cells can then differentiate into remyelinating oligodendrocytes (Gensert and Goldman, 1997). Importantly, the proliferation and migration of OPCs is proportional to the inflammatory response (Glezer et al., 2006) and corresponds to the peak of inflammation (Ben-Hur et al., 2003). Here, we observed indeed that an enhanced proliferation of OPCs in the corpus callosum of mice treated with mCSF, reflective of the higher number of microglial cells as well as of the expression level of TLR2.

Despite that inflammation has been seen as a noxious event in MS, paradoxically we have confirmed that stimulating the microglial response in a timely manner is protective. Thus, the next question was how remyelination is promoted and what factors may play a role.

Previous studies have identified factors involved in oligodendrocytes viability and in remyelination. Of note among these factors, is IGF-1, one of the most studied growth factors during remyelination processes. It is expressed by glial cells including microglia (Matsushima and Morell, 2001), stimulates oligodendrocyte survival, development and differentiation, and the production of myelin sheaths (Mason et al., 2000b; Clemente et al., 2013). Importantly, we observed that its gene expression levels increased following mCSF treatment in cuprizone-intoxicated mice and it was found to parallel the proliferation of OPCs within lesions, promoting the recovery of oligodendrocytes (Mason et al., 2000a), without preventing the accumulation of microglia (Mason et al., 2000b). The increase expression of IGF-1 may consequently be a key mechanism to protect oligodendrocytes or stimulate their differentiation from OPCs in mice that received mCSF earlier in the demyelinating course of events. However the contribution of microglia in this process is still not fully understood (Wlodarczyk et al., 2017). In response to dietary cuprizone, massive immune response is localized in the demyelinated corpus callosum where a subpopulation of CD11c+ microglial cells was previously reported (Remington et al., 2007). These cells were associated to the expression of IGF-1 in a model of EAE indicating that they may have a protective role in the disease (Wlodarczyk et al., 2015). mCSF promoting microglial proliferation might consequently modulate the increase of this subpopulation of cells and release of IGF-1. Although after 4 weeks of mCSF treatment we observed very positive results on the myelin preservation of the corpus callosum, previously it was shown that a persistent stimulation of the immune response is detrimental in MS (Yong, 2010). Thus, we decided to test different regimens of mCSF treatment. Indeed, the drug was injected during the first weeks of the diet as well as the end of the diet. Interestingly, the increased expression levels of IGF-1 in the corpus callosum remained sustained over time when mCSF injections were limited to the initial phases of disease progression, resulting in similar level of remyelination as in mice treated for the entire duration of the diet. Importantly, when mCSF was given at in the remyelination phase, the drug did not have any beneficial effects on the remyelination of the corpus callosum. These data indicate that mCSF should be administered in a very precise phase of MS progression, the initial phases, to exacerbate its protective and beneficial role. However, the increase of IGF-1 alone might not be sufficient as IGF-1 infusion did not alter the course of the disease in the EAE model (Cannella et al., 2000; Genoud et al., 2005).

As a persistent stimulation of the immune response is detrimental in MS (Yong, 2010), and although we obtained positive results with a bi-weekly administration of mCSF, we decided to test different regimens of the treatment. Indeed, the drug was injected once a week during the first 2 or 3 week of the diet as well as the end of the diet (data not shown). Interestingly, only two doses of mCSF at the beginning of the diet are sufficient to obtain similar beneficial effect of the drug (compared with original protocol). The increased expression levels of IGF-1 in the corpus callosum remained sustained over time when mCSF injections were limited to the initial phases of disease progression, resulting in similar myelin levels as in mice treated for the entire duration of the diet. These data indicate that the timing of mCSF administration is critical. Although the 4-weeks regimen was beneficial in the original experiment, such a treatment did not reach significant level in the subsequent protocol where the cytokine was administered for a period of 5 weeks. Despite a similar tendency in both protocols, differences are often quite subtle and may explain the lack of significant differences between groups of animals. Sensitivity to the cytokine may also differ during a longer period and such effects may be taking place between weeks 4 and 5. Further experiments will be needed to clarify such possible mechanisms and whether the CSFR1 is at play. Be that as it may, administering the cytokine early and for a short period of time seems the best strategy to protect the myelin.

mCSF acts through complementary mechanisms, stimulating the proliferation of microglial cells and its phagocytic activity, essential for the removal of myelin debris which could inhibit OPCs differentiation (Kotter et al., 2001; Franklin and Kotter, 2008). Indeed, following demyelination, oligodendrocytes die, leaving behind myelin debris which is then removed by phagocytic cells. mCSF treatment boosts phagocytosis, enhancing the expression of TLR2 (Figure 4), *TREM2* (Figure 3C) or lysozyme (Matsushima and Morell, 2001). TLR2 is a transmembrane receptor that is strongly expressed in microglial cells during diseases (Glezer et al., 2007) and it is a reliable marker of activated microglia (Laflamme et al., 2001). Importantly, it is involved in phagocytic activity of innate immune cells (Fang et al., 2014) and the TLR2 cascade might be crucial for recovery after injury (Kigerl et al., 2007). Indeed, TLR signaling is essential for orchestrating the innate immune response, eventually leading to myelin clearance (Boivin et al., 2007). Another essential player is TREM2, a membrane-bound receptor expressed by microglia and infiltrating macrophages (Piccio et al., 2007) and it is known to reduce inflammatory response and to be implicated in the phagocytic activities of immune cells, inducing myelin and amyloid beta removal (Piccio et al., 2008; Boissonneault et al., 2009; Smith et al., 2013). Interestingly, TREM2 is up-regulated in EAE model during both the early and chronic inflammatory phases of pathology. Its blockade during the effector phase of EAE exacerbates pathology, increasing immune cell infiltration and demyelination (Piccio et al., 2007) underling why the clearance of myelin debris is critical to allow OPC differentiation and remyelination (Kotter et al., 2006). Indeed, delayed myelin clearance is also associated with delayed OPC differentiation (Kotter et al., 2005). We also observed irregular myelin fibers as well as a burden of myelin debris in the corpus callosum of CSF1R conditional knockout mice. Importantly, the area of interest was largely devoid of CD68^+^ cells, a frequently used marker to identify phagocytosing cells. On the contrary, mCSF-treated mice exhibited an improved recovery of myelin fibers as well as a better clearance. Further electron microscopy analyses will be necessary to confirm our preliminary observations in the cuprizone model. Additionally, in the knocked-out model, we report a reduced number of Olig2^+^ cells—that might be reflective of mature oligodendrocyte loss—along with reduced expression of *PDGFRα* (data not shown). These results suggest that the lack of the mCSF receptor may affect oligodendrocyte proliferation and survival, however deeper studies are needed to corroborate our observations. The proper elimination of myelin debris might be beneficial while stimulating a regenerative environment (Neumann et al., 2009).

We cannot exclude the possible contribution of infiltrating monocytes to the beneficial effects of mCSF, although there are many data that strongly suggest otherwise. First, the cytokine did not influence significantly the number of infiltrating cells in chimeric mice, while it strongly triggered proliferation of resident microglia. Second, the model used to delete CSF1R using the CX3CR1-cre-ERT2 system is based on the tamoxifen regimen that selectively affects long-lived microglia with negligible effects on shorter-lived blood monocytes (Yona et al., 2013; Peng et al., 2016; Fonseca et al., 2017). Third, we previously reported a critical role for CX3CR1 signaling in resident microglia, clearing myelin debris to improve remyelination in the same cuprizone model without affecting infiltrating blood monocytes (Lampron et al., 2015). In the same study, deletion of CCR2 strongly inhibited infiltration of these innate immune cells in demyelinating regions, but this did not modulate significantly demyelination and remyelination (Lampron et al., 2015). Consequently, we believe that the strong expression of CD68 by resident microglia is indicative of their strong phagocytic potential in wild-type mice while this phenomenon is essentially inexistent in the CSF1R-loxP-CX3CR1-cre/ERT2 mice treated with tamoxifen.

In conclusion, we provide here a novel therapeutic avenue for the use of mCSF that is now becoming a very critical cytokine to stimulate beneficial physiological functions of microglia. It triggers their proliferation, their expression of scavenger receptors and their phagocytic response toward myelin debris, which are all associated with survival of OPCs as well as proper remyelination and repair. On the contrary, selective and conditional elimination of its receptor CSF1R in microglia impairs this natural innate immune mechanism causing aberrant myelin debris clearance, which interferes with proper repair processes. Since the current immunomodulatory therapies for MS have failed to prevent patients from entering the progressive phase of the disease (Stys et al., 2012), mCSF would be an ideal target for a clinical trial in individuals diagnosed with primary and secondary progressive MS.

## Summary

mCSF treatment reduced myelin loss in an animal model of MS, whereas conditional deletion of its receptor in microglia caused aberrant myelin debris accumulation. mCSF therefore plays a key role in stimulating myelin clearance, proper remyelination and myelin repair.

## Author Contributions

NL drove the project, did the analyses and most of the manipulations, coordinated the figure preparation and was involved in writing and revising the manuscript. GC coordinated, prepared and assembled all figures, did graphical and statistical analyses and wrote the manuscript together with NL. PP took all confocal images and did some manipulations. YS and JB did some histological preparations. M-KS-P conducted the EM experiments. M-ÈT supervised the EM experiments and conducted analysis. SR formulated the study concept, supervised the project in its entirety and was involved in the writing and revising the manuscript.

## Supplementary Material

FIGURE S1 The Supplementary Material for this article can be found online at: https://www.frontiersin.org/articles/10.3389/fncel.2018.00178/full#supplementary-material
Food intake and body weight throughout cuprizone-supplemented diet. Mice were fed with normal chow or cuprizone-supplemented chow (2 mg/kg) for 5 week and injected two times/week with mCSF (40 μg/kg) or saline (0.9%) during the four first weeks of diet. **(A)** Fifty gram of ground food, normal or cuprizone-supplemented, was given to mice every other day and the leftover was calculated as grams of food ingested per day, divided by the number of mice in the cage (*n* = 8 mice/group). **(B)** Body weight of mice was recorded every 2–3 days throughout the protocol (*n* = 8–10 mice/group). Values are expressed as means ± SEM.

FIGURE S2 Effect of mCSF administration on cuprizone-induced rostral demyelination. **(A)** Representative photomicrograph showing myelin staining (Black Gold II) in the rostral corpus callosum of mice fed with normal or cuprizone-supplemented chow for 5 weeks and injected two times/week with mCSF (40 μg/kg) or saline (0.9%). Scale bar: **A**: 150 μm.

FIGURE S3 Prolonged administration of mCSF following the cuprizone supplement-diet did not impact remyelination. **(A)** Timeline of mCSF administration during 5 weeks of cuprizone-supplemented diet plus one 1 week of normal diet. **(B)** Representative photomicrograph showing myelin staining (Black Gold II) in the medial corpus callosum of mice fed with normal or cuprizone-supplemented chow for 5 weeks and injected two times/week with mCSF (40 μg/kg) or saline (0.9%) during the entire protocol. **(C)** Quantification of demyelination, presented as percentage of area occupied by the staining, measured in the corpus callosum. Values are expressed as means ± SEM.

FIGURE S4 Administration of mCSF during cuprizone supplement-diet promotes Olig2 proliferation. **(A,B)** Representative photomicrographs of olig2-immunoreactive staining **(A)** and its quantification **(B)** in the corpus callosum of mice fed with normal or cuprizone-supplemented chow for 5 weeks and injected twice a week with either saline or mCSF (40 μg/kg) during the first 4 weeks of diet and finally sacrificed 1 week after the 5 weeks of diet. Values are expressed as means ± SEM. Statistical analyses were performed using unpaired *t*-test. *p < 0.05, significantly different from the group that received the cuprizone-supplemented chow + mCSF. *n* = 4–5 mice. Scale bar: **A**: 150 μm. Abbreviations: cc, corpus callosum; d, days; mCSF, macrophage colony stimulating factor; Olig2, oligodendrocyte transcription factor 2; w, weeks.

FIGURE S5 mCSF does not promote recruitment of peripheral immune cells but boosts cell proliferation in the corpus callosum. **(A,B)** Representative images of GFP infiltrating cells in the corpus callosum of chimeric mice fed with cuprizone-supplemented chow for 5 weeks and injected twice a week with either saline **(A)** or mCSF (40 μg/kg) **(B)** during the first 4 weeks of diet. **(C)** Graph representatives of the number of GFP+ cells in the CC counted by stereology. Values are expressed as means ± SEM. *n* = 6–8 mice. Statistical analyses were performed using an unpaired t-test. Scale bar: **A,B**: 100 μm; Abbreviations: cc, corpus callosum; Cup, cuprizone; GFP, green fluorescent protein; mCSF, macrophage colony stimulating factor.

## Conflict of Interest Statement

The authors declare that the research was conducted in the absence of any commercial or financial relationships that could be construed as a potential conflict of interest.
