# Supplementary figures and images for: mCSF-Induced Microglial Activation Prevents Myelin Loss and Promotes Its Repair in a Mouse Model of Multiple Sclerosis
Source: Front Cell Neurosci. 2018 Jul 3;12:178. doi: 10.3389/fncel.2018.00178 (PMC6037698; doi:10.3389/fncel.2018.00178)

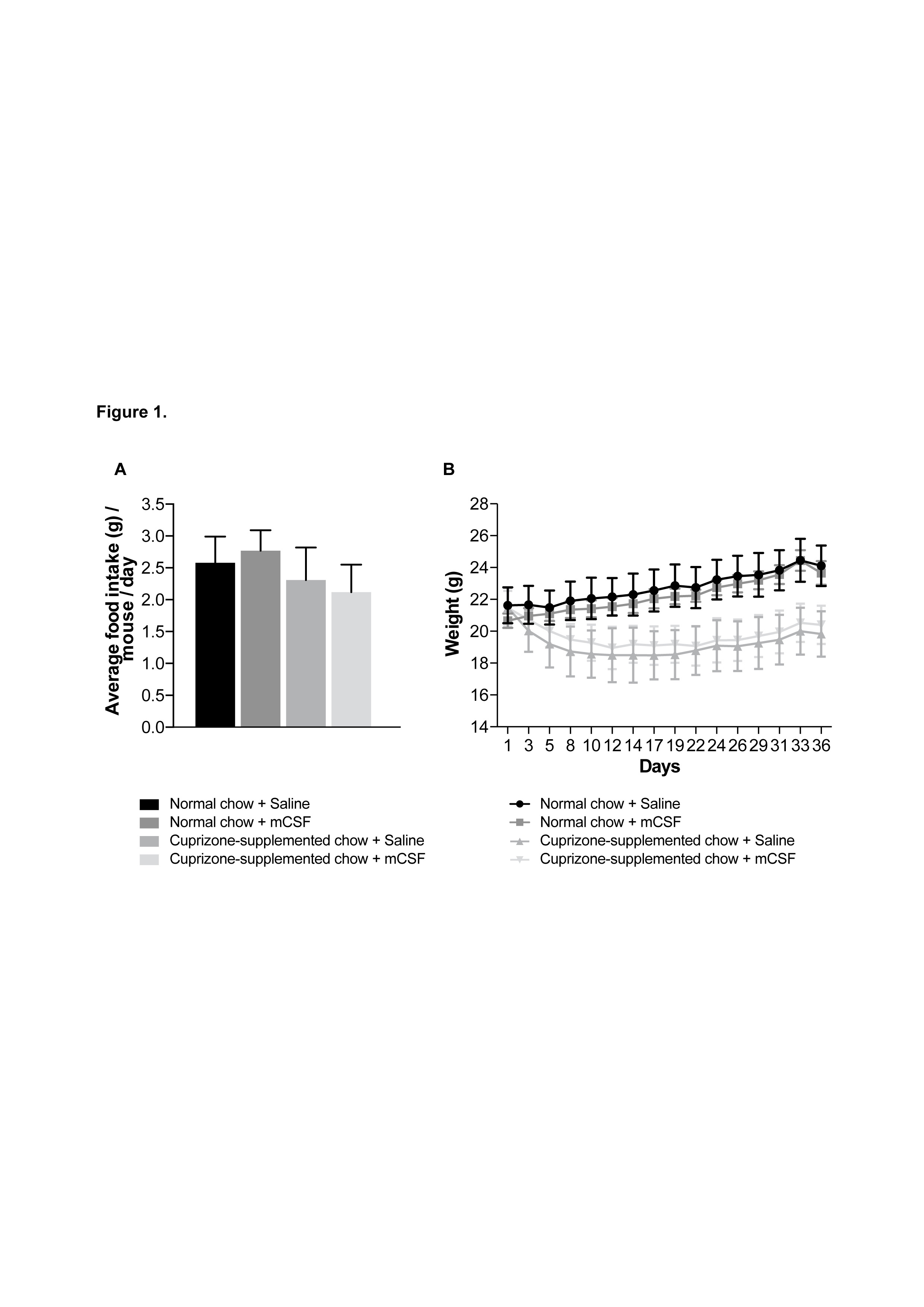

Supplement: FIGURE S1 — The Supplementary Material for this article can be found online at: https://www.frontiersin.org/articles/10.3389/fncel.2018.00178/full#supplementary-material Food intake and body weight throughout cuprizone-supplemented diet. Mice were fed with normal chow or cuprizone-supplemented chow (2 mg/kg) for 5 week and injected two times/week with mCSF (40 μg/kg) or saline (0.9%) during the four first weeks of diet. (A) Fifty gram of ground food, normal or cuprizone-supplemented, was given to mice every other day and the leftover was calculated as grams of food ingested per day, divided by the number of mice in the cage (n = 8 mice/group). (B) Body weight of mice was recorded every 2–3 days throughout the protocol (n = 8–10 mice/group). Values are expressed as means ± SEM. [file Image_1.JPEG]

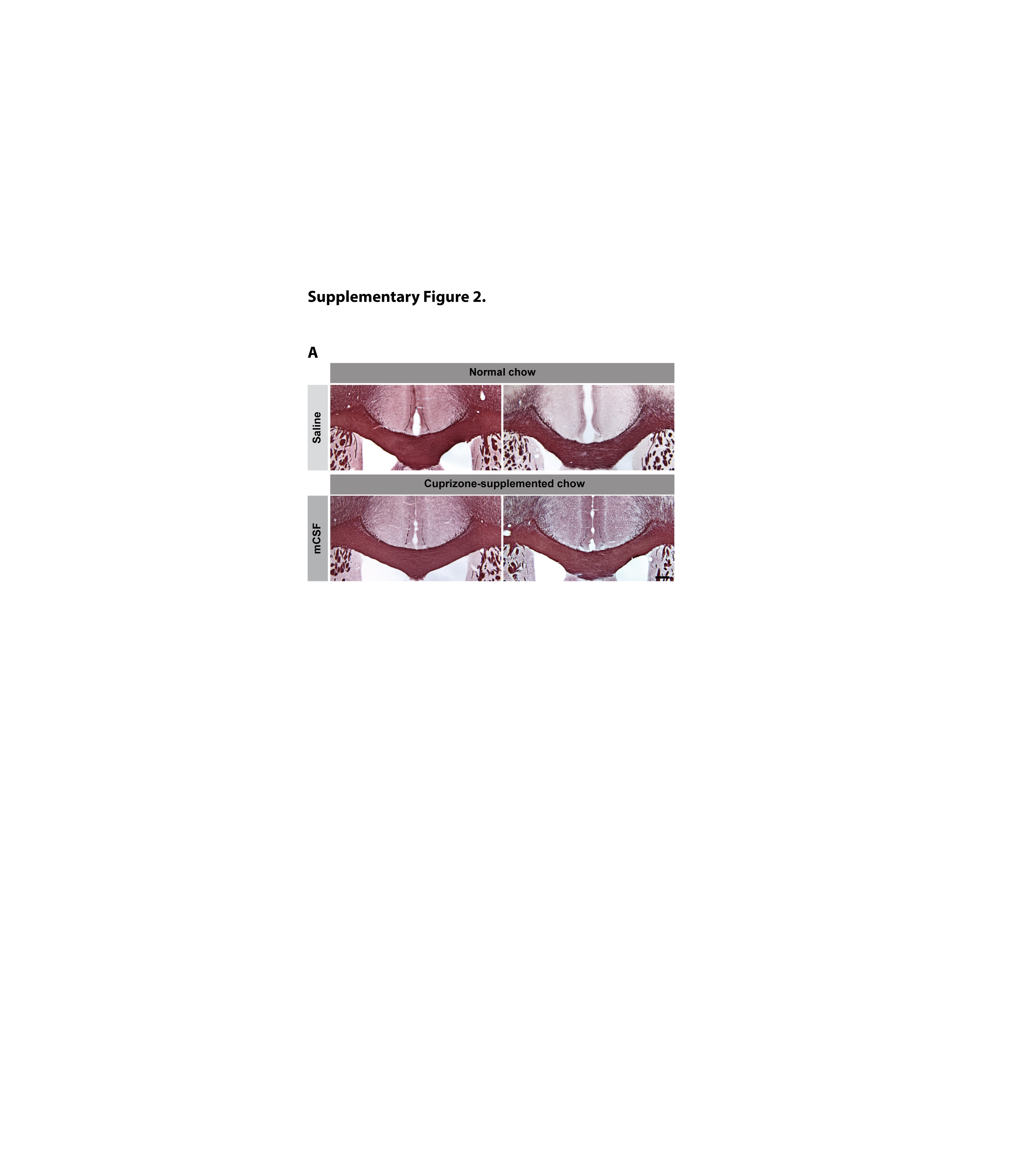

Supplement: FIGURE S2 — Effect of mCSF administration on cuprizone-induced rostral demyelination. (A) Representative photomicrograph showing myelin staining (Black Gold II) in the rostral corpus callosum of mice fed with normal or cuprizone-supplemented chow for 5 weeks and injected two times/week with mCSF (40 μg/kg) or saline (0.9%). Scale bar: A: 150 μm. [file Image_2.JPEG]

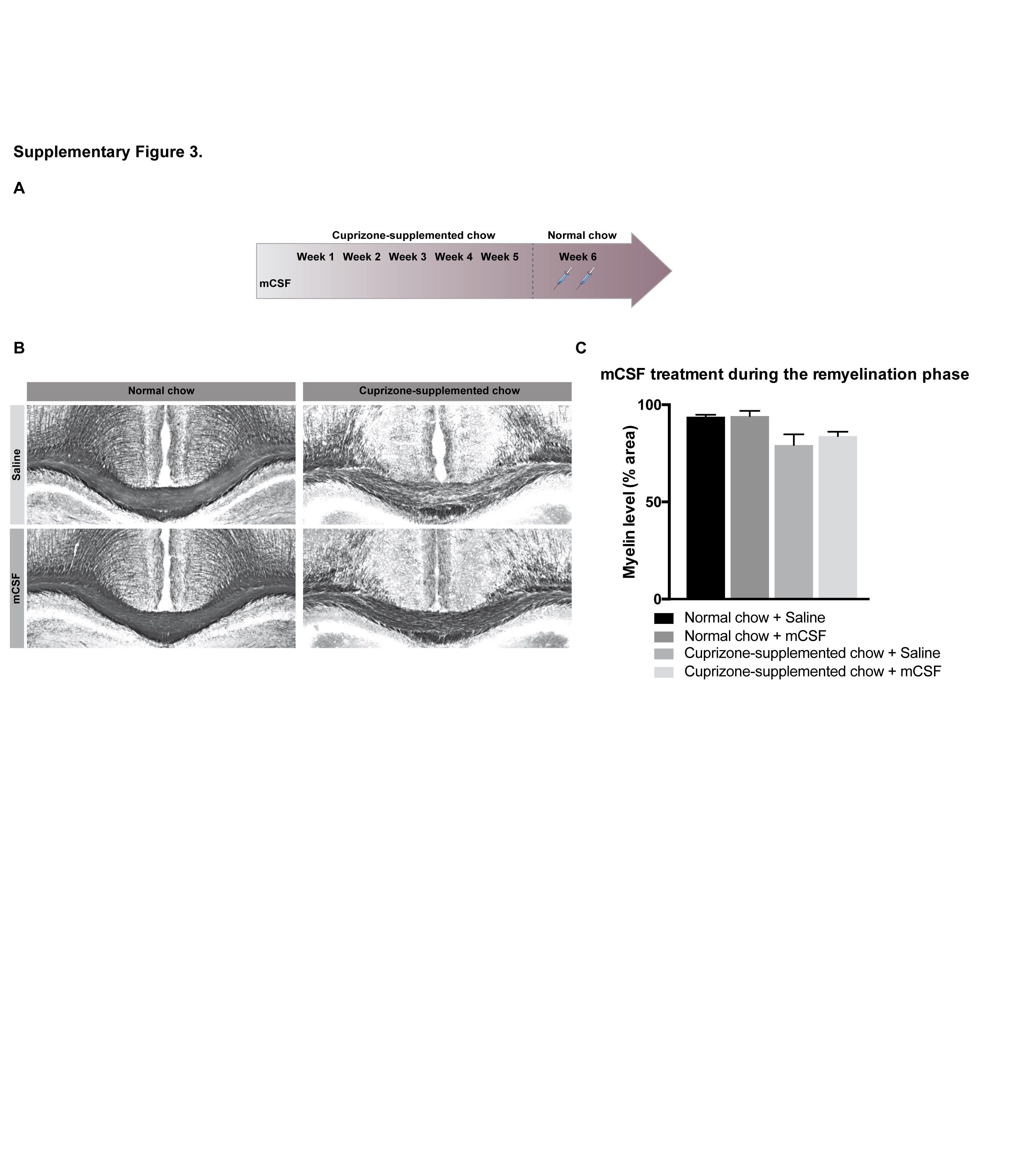

Supplement: FIGURE S3 — Prolonged administration of mCSF following the cuprizone supplement-diet did not impact remyelination. (A) Timeline of mCSF administration during 5 weeks of cuprizone-supplemented diet plus one 1 week of normal diet. (B) Representative photomicrograph showing myelin staining (Black Gold II) in the medial corpus callosum of mice fed with normal or cuprizone-supplemented chow for 5 weeks and injected two times/week with mCSF (40 μg/kg) or saline (0.9%) during the entire protocol. (C) Quantification of demyelination, presented as percentage of area occupied by the staining, measured in the corpus callosum. Values are expressed as means ± SEM. [file Image_3.jpg]

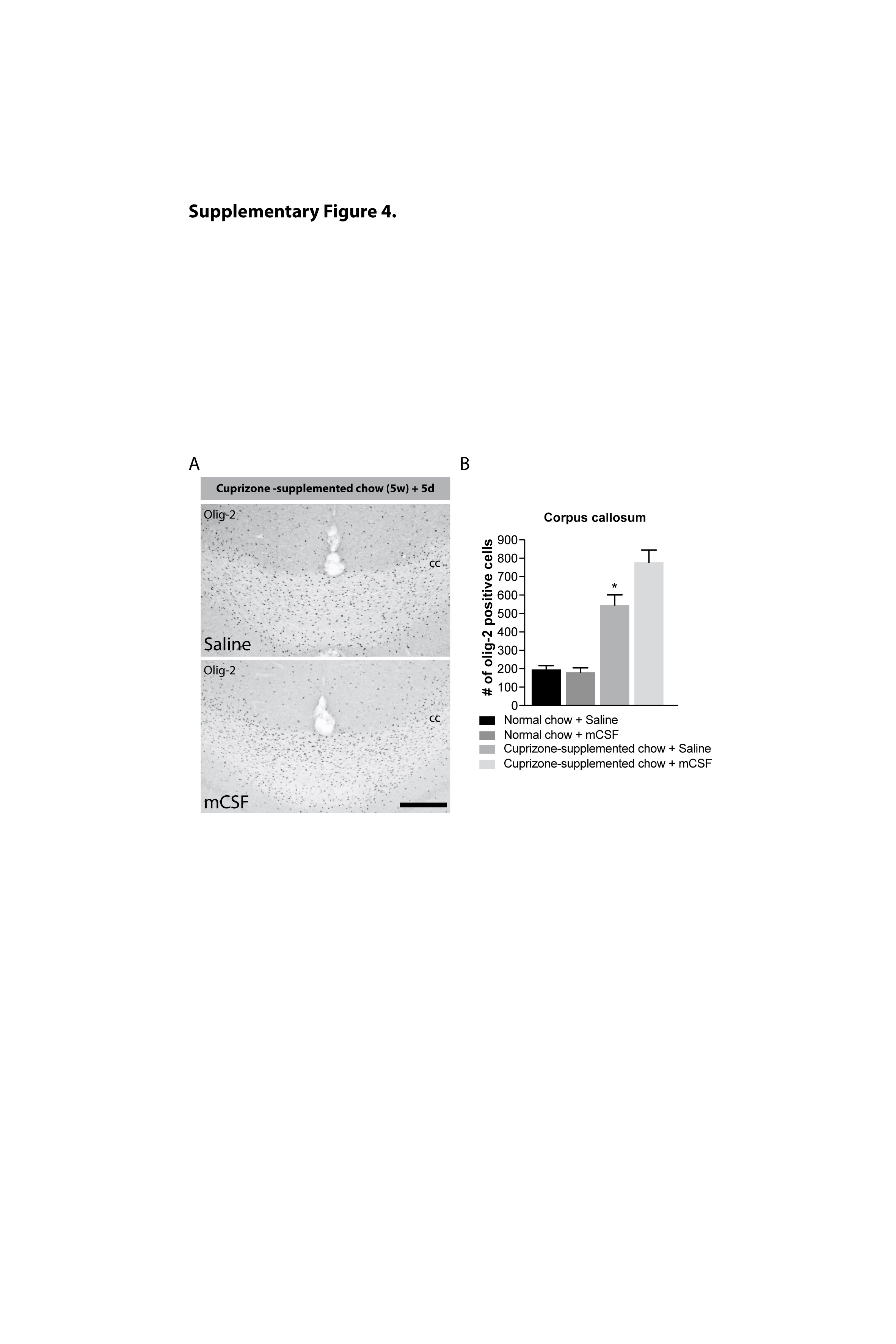

Supplement: FIGURE S4 — Administration of mCSF during cuprizone supplement-diet promotes Olig2 proliferation. (A,B) Representative photomicrographs of olig2-immunoreactive staining (A) and its quantification (B) in the corpus callosum of mice fed with normal or cuprizone-supplemented chow for 5 weeks and injected twice a week with either saline or mCSF (40 μg/kg) during the first 4 weeks of diet and finally sacrificed 1 week after the 5 weeks of diet. Values are expressed as means ± SEM. Statistical analyses were performed using unpaired t-test. *p < 0.05, significantly different from the group that received the cuprizone-supplemented chow + mCSF. n = 4–5 mice. Scale bar: A: 150 μm. Abbreviations: cc, corpus callosum; d, days; mCSF, macrophage colony stimulating factor; Olig2, oligodendrocyte transcription factor 2; w, weeks. [file Image_4.JPEG]

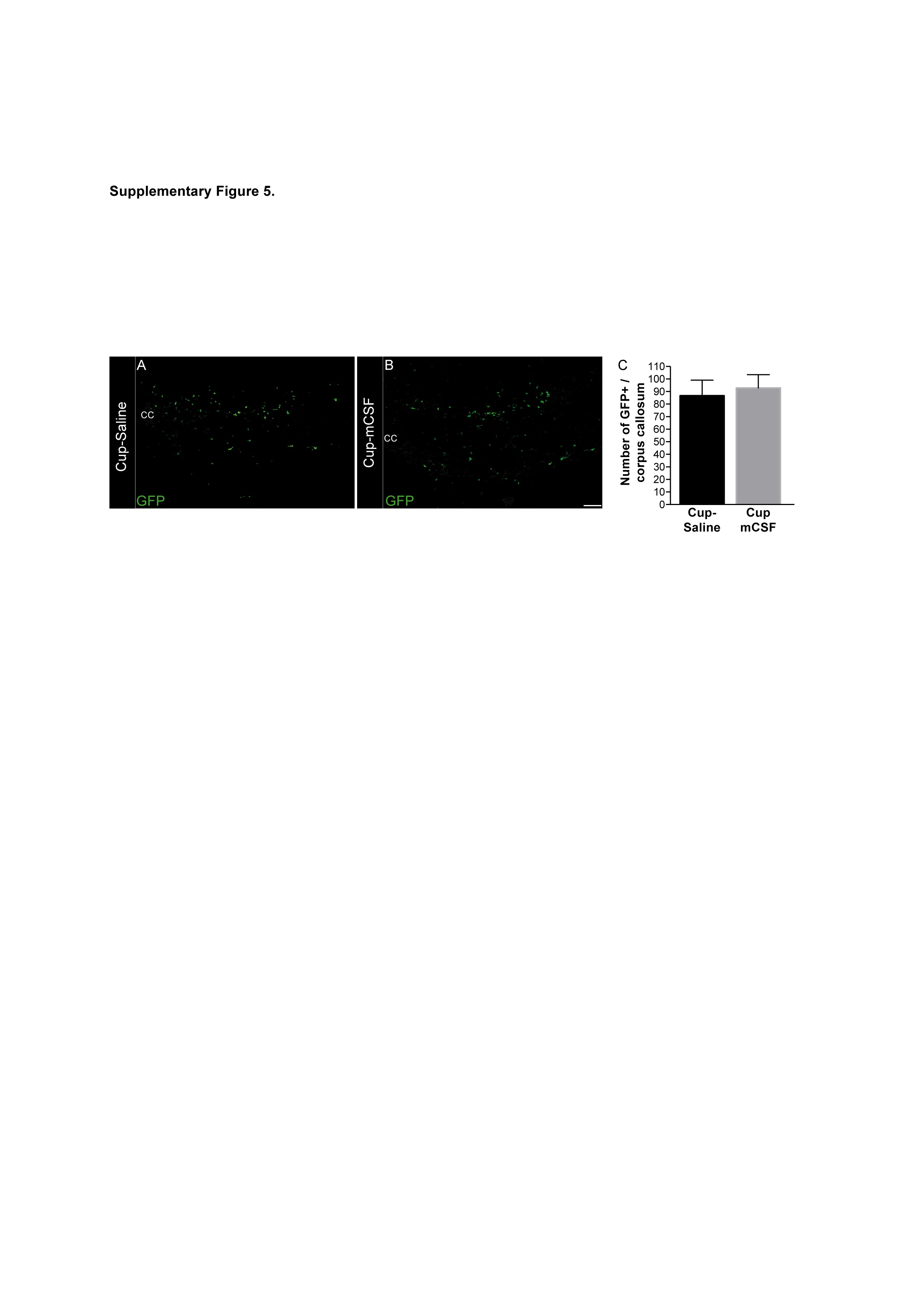

Supplement: FIGURE S5 — mCSF does not promote recruitment of peripheral immune cells but boosts cell proliferation in the corpus callosum. (A,B) Representative images of GFP infiltrating cells in the corpus callosum of chimeric mice fed with cuprizone-supplemented chow for 5 weeks and injected twice a week with either saline (A) or mCSF (40 μg/kg) (B) during the first 4 weeks of diet. (C) Graph representatives of the number of GFP+ cells in the CC counted by stereology. Values are expressed as means ± SEM. n = 6–8 mice. Statistical analyses were performed using an unpaired t-test. Scale bar: A,B: 100 μm; Abbreviations: cc, corpus callosum; Cup, cuprizone; GFP, green fluorescent protein; mCSF, macrophage colony stimulating factor. [file Image_5.JPEG]
